# Serum neutrophil gelatinase-associated lipocalin and lactate level during surgery predict acute kidney injury and early allograft dysfunction after liver transplantation

**DOI:** 10.1038/s41598-023-34372-9

**Published:** 2023-05-27

**Authors:** Hyeyeon Cho, Ji-Yoon Jung, Hyun-Kyu Yoon, Seong-Mi Yang, Ho-Jin Lee, Won Ho Kim, Chul-Woo Jung, Kyung-Suk Suh

**Affiliations:** 1 Department of Anesthesiology and Pain Medicine, Seoul National University Hospital, Seoul National University College of Medicine, 101 Daehak-Ro, Jongno-Gu, Seoul, 03080 Republic of Korea; 2 Department of Surgery, Seoul National University College of Medicine, Seoul, Republic of Korea

**Keywords:** Medical research, Biomarkers, Predictive markers, Liver, Liver diseases

## Abstract

Early allograft dysfunction (EAD) and acute kidney injury (AKI) are common and clinically important complications after liver transplantation. Serum lactate level at the end of surgery could predict EAD and neutrophil gelatinase-associated lipocalin (NGAL) is known as a biomarker for AKI after liver transplantation. The authors investigated whether the combination of these two laboratory tests could be used as an early predictor of these two complications of EAD and AKI. We reviewed cases undergoing living donor liver transplantation (n = 353). Lactate-adjusted NGAL level, a combination of these two predictors, was calculated as the sum of each value multiplied by the odds ratio for EAD or AKI. We evaluated whether this combined predictor at the end of surgery is significantly associated with both postoperative AKI or EAD. We compared the area under the receiver operating characteristic curve (AUC) between our multivariable regression models with and without NGAL, lactate, or lactate-adjusted NGAL. NGAL, lactate and lactate-adjusted NGAL are significant predictors for EAD and AKI. The regression model for EAD or AKI including lactate-adjusted NGAL showed a greater AUC (for EAD: odds ratio [OR] 0.88, 95% confidence interval [CI] 0.84–0.91; for AKI: OR 0.89, 95% CI 0.85–0.92) compared to the AUC of the models including lactate (for EAD: OR 0.84, 95% CI 0.81–0.88; for AKI: OR 0.79, 95% CI 0.74–0.83) or NGAL alone (for EAD: OR 0.82, 95% CI 0.77–0.86; for AKI: OR 0.84, 95% CI 0.80–0.88) or the model without lactate or NGAL (for EAD: OR 0.64, 95% CI 0.58–0.69, for AKI: OR 0.75, 95% CI 0.70–0.79). In conclusion, lactate-adjusted NGAL level at the end of surgery could be a reliable combined laboratory predictor for postoperative EAD or AKI after liver transplantation with a greater discriminative ability than lactate or NGAL alone.

## Introduction

Among the novel serum or urine biomarkers to predict acute kidney injury (AKI) after liver transplantation in patients with liver cirrhosis, neutrophil gelatinase-associated lipocalin (NGAL) along with cystatin C has been studied in the largest volume of studies^1^. Although NGAL is a known valid marker to detect AKI early, it could not independently predict early allograft dysfunction (EAD) or 30-day liver-related mortality^2^.

Lactic acid is a metabolite and is metabolized predominantly in the liver. In liver transplantation, a low clearance of serum lactate after graft reperfusion is considered to be associated with impaired graft function. A previous study reported that an elevated arterial lactate concentration at the end of liver transplantation surgery could be an early predictor of posttransplant graft dysfunction^3^. Its prognostic value for the postoperative liver function was also demonstrated in an elective hepatectomy^4^. Hyperlactatemia at the end of liver resection surgery was an independent risk factor for postoperative morbidities.

Among the complications after liver transplantation, AKI and EAD are particularly relevant due to their high incidence and clinical implication^5–8^. AKI is associated with long-term graft failure or all-cause mortality^9^ and also with long-term renal dysfunction and the development of chronic kidney disease. Therefore, a balanced combination of laboratory predictors for AKI and EAD could be a simple and useful prognostic factor for patients undergoing liver transplantation compared to any single prognostic laboratory parameter.

Therefore, in this retrospective observational study, we sought to investigate whether serum NGAL, lactate concentration at the end of surgery or their combination have significant predictive accuracy and the discriminative ability for EAD and AKI. We also evaluated whether their combination has an additional predictive ability for EAD or AKI by comparing the predictive ability of the multivariable regression model with and without NGAL or lactate or their combination.

## Methods

This study was reported according to the STROBE statement checklist for an observational study^10^. We obtained approval for our retrospective cohort study from the Institutional Review Board of Seoul National University Hospital (H-2205-084-1324). We received a waiver of written informed consent from the board, considering the retrospective nature of our study. All methods were performed following the relevant guidelines and regulations.

We reviewed the institutional electronic database of 450 consecutive patients who underwent deceased or living donor liver transplantation at our tertiary care university hospital between January 2019 and April 2022. The patients with preoperative renal dysfunction (n = 24), missing baseline or outcome parameters (n = 21), retransplantation due to graft failure after previous transplantation (n = 4), and deceased donor transplantation (n = 48) were excluded. The remaining 353 patients were included in our analysis.

We extracted demographic or perioperative data previously reported to have an association with postoperative EAD and AKI after liver transplantation from our institutional electronic medical record database (Table 1)^5–8,11,12^. Early allograft dysfunction was defined when one or more of the following are present within the first 7 postoperative days: total bilirubin ≥ 10 mg/dL, prothrombin time: international normalized ratio ≥ 1.6^13^. We determined AKI by the Kidney Disease Improving Global Outcomes criteria, which was diagnosed according to the maximal change in serum creatinine level during the first seven postoperative days (Stage 1: 1.5–1.9; stage 2: 2–2.9; stage 3: more than threefold increase from baseline value or increase in serum creatinine to ≥ 4.0 mg/dL or initiation of renal replacement therapy)^14,15^. The most recent serum creatinine value measured before surgery was collected as a baseline.

**Table 1:** Patient characteristics and perioperative parameters in all patients (n = 353).

Characteristic	All patients
Demographic data	
Age, years	58 (52–63)
Female, n	117 (33.1)
Body-mass index, kg/m^2^	25.3 (23.0–28.2)
Background medical status	
Hypertension, n	79 (22.4)
Diabetes mellitus, n	106 (30.0)
Alcoholic liver cirrhosis, n	88 (24.9)
HBV hepatitis, n	188 (53.3)
HCV hepatitis, n	25 (7.1)
Hepatocellular carcinoma, n	200 (56.7)
Cholestatic disease, n	29 (8.2)
Preoperative hemoglobin, g/dl	10.5 (8.8–12.6)
Preoperative serum albumin level, mg/dl	3.2 (2.8–3.7)
MELD score	10.5 (8.0–15.7)
CTP score	7 (5–9)
Child class, n (A/ B/ C)	157 (44.5)/122 (34.6)/74 (21.0)
Hepatorenal syndrome	–
Previous abdominal surgery, n	96 (27.2)
Preoperative LVEF, %	
Preoperative beta-blocker, n	39 (11.0)
Preoperative diuretics, n	91 (25.8)
Donor/ graft factors	
Age, years	34 (25–43)
Estimated GRWR	1.13 (0.93–1.33)
Operation and anesthesia details	
Operation time, hour	395 (336–480)
Cold ischemic time, min	100 (80–134)
Warm ischemic time, min	33 (27–42)
Crystalloid administration	4550 (3525–6450)
20% albumin, ml	500 (300–700)
Bleeding and transfusion amount	
pRBC transfusion, units	4 (0–10)
FFP transfusion, units	1 (0–7)
Platelet concentrate, units	0 (0–1)
Blood loss per body weight, ml/kg	2300 (1400–4800)

The values are expressed as the median [interquartile range] or number (%).

*HBV* hepatitis B virus, *HCV* hepatitis C virus, *MELD score* model for end-stage liver disease score, *CTP score* Child–Turcotte–Pugh score, *LVEF* left ventricular ejection fraction, *GRWR* graft-to-recipient weight ratio, *pRBC* packed red blood cells, *FFP* fresh frozen plasma.

GRWR was calculated by dividing the weight of the liver graft by the weight of the recipient, multiplied by 100 to express it as a percentage. For example, a GRWR of 0.8 means that the weight of the graft is 0.8% of the recipient's body weight.

Serum lactate level at the end of surgery was used because previous studies reported the prognostic value at that time^3,4^. Serum NGAL level was measured twice during surgery—at baseline and at the end of surgery. The level at the end of surgery was used in our analysis because the baseline NGAL level was not significantly different between the patients with and without AKI or EAD in our preliminary analysis. We defined the combination of lactate and NGAL as lactate-adjusted NGAL, which was calculated according to the following equation for EAD and AKI, separately. It was calculated as the sum of each measured value multiplied by each odds ratio for EAD or AKI calculated by our multivariable logistic regression analysis.

$$ \begin{aligned} & {\text{Lactate-adjusted NGAL for EAD }} = { 1}.{41}*{\text{lactate }} + { 1}.0{3}*{\text{NGAL}} \\ & {\text{Lactate-adjusted NGAL for AKI }} = { 1}.0{2}*{\text{lactate }} + { 1}.{27}*{\text{NGAL}} \\ \end{aligned} $$

### Statistical analysis

Before statistical analyses, we determined the normality of each continuous variable using the Shapiro–Wilk test. Continuous data are reported as the median (25 and 75 percentiles) and were compared by the Mann–Whitney *U* test. We compared incidence data by or χ^2^ test or Fisher’s exact test according to their expected counts. Baseline characteristics or outcome data were missing in 4.5% of records. We excluded these missing cases before the main statistical analysis. Baseline characteristics did not differ significantly between cases with and without missing parameters in our preliminary analysis.

The followings are the main analyses of our study. Firstly, we performed binary multivariable logistic regression analysis to investigate the association of serum NGAL and lactate level with the risk of postoperative EAD and AKI after liver transplantation, separately. All covariates previously reported as the risk factors for EAD and AKI were included. No variable selection process was used in the regression analysis. We evaluated our regression model’s calibration and discrimination by the Hosmer–Lemeshow goodness of fit test and c-statistics, respectively.

Secondly, to compare the diagnostic value of serum NGAL, lactate level and their combination—lactate-adjusted NGAL for our clinical outcomes, the area under the receiver operating characteristics curve (AUC) for each logistic regression analysis was compared. AUCs of multivariable-adjusted regression models with and without NGAL, lactate and lactate-adjusted NGAL level were compared to investigate whether the addition of NGAL, lactate or lactate-adjusted NGAL level to our multivariable model could increase the discriminative ability for EAD or AKI. DeLong’s method was used to compare different AUCs^16^. To determine a meaningful cutoff of serum lactate-adjusted NGAL, Youden’s index where the sum of sensitivity and specificity is maximal was used for EAD and AKI, respectively^17^.

Thirdly, we drew cubic spline function curves to investigate the multivariable-adjusted relationship of the serum NGAL, lactate and lactate-adjusted NGAL level as a continuous variable with the risk of EAD and AKI.

Fourthly, we performed propensity score matching between the two lactate-adjusted NGAL groups to adjust the potential confounding effect of the baseline patient characteristics along with anesthesia and surgery-related parameters. Matching was performed for the two lactated-adjusted NGAL groups for EAD and AKI, respectively. The following variables were used for matching: patient demographics, past medical history of hypertension, diabetes mellitus, baseline laboratory values including hemoglobin, serum albumin level, Models for end-stage liver disease (MELD) score, Child classification, history of previous abdominal surgery, baseline left ventricular ejection fraction, preoperative medication of beta-blocker, diuretics, estimated graft-recipient body-weight ratio, operation time, cold and warm ischemic time, the amount of intraoperative crystalloids and albumin administration, and intraoperative estimated blood loss. The caliper width of 0.2 standard deviations of the logit-transformed propensity score was used. Then we compared the clinical outcomes between the two matched groups.

We presented data as median (interquartile range) or number (%). All P values are calculated for two-sided hypothesis testing, and statistical significance was determined at the significance level of 0.05. Multiple comparisons were adjusted by Bonferroni correction. Stata 15.1 (StataCorp, College Station, TX, USA) was used for our statistical analyses.

### Ethics statement

We obtained approval for our retrospective cohort study from the Institutional Review Board of Seoul National University Hospital (H-2205-084-1324). We received a waiver of written informed consent from the board, considering the retrospective nature of our study.

## Results

Supplemental Figure S1 shows the inclusion and exclusion of our study cohort. After excluding cases with exclusion criteria described in Methods as well as deceased donor transplantation (n = 48), our final dataset included 353 living donor liver transplant cases. The incidence of AKI was 24.6% (n = 87/353) in our retrospective cohort with stage 1 (n = 56, 15.9%), stage 2 (n = 19, 5.4%) and stage 3 (n = 15, 3.7%). The incidence of EAD was 6.2% (n = 22/353). There was no case with the small-for-size syndrome. Table 1 shows the baseline characteristics and perioperative parameters of our study cohort.

**Figure 1:**
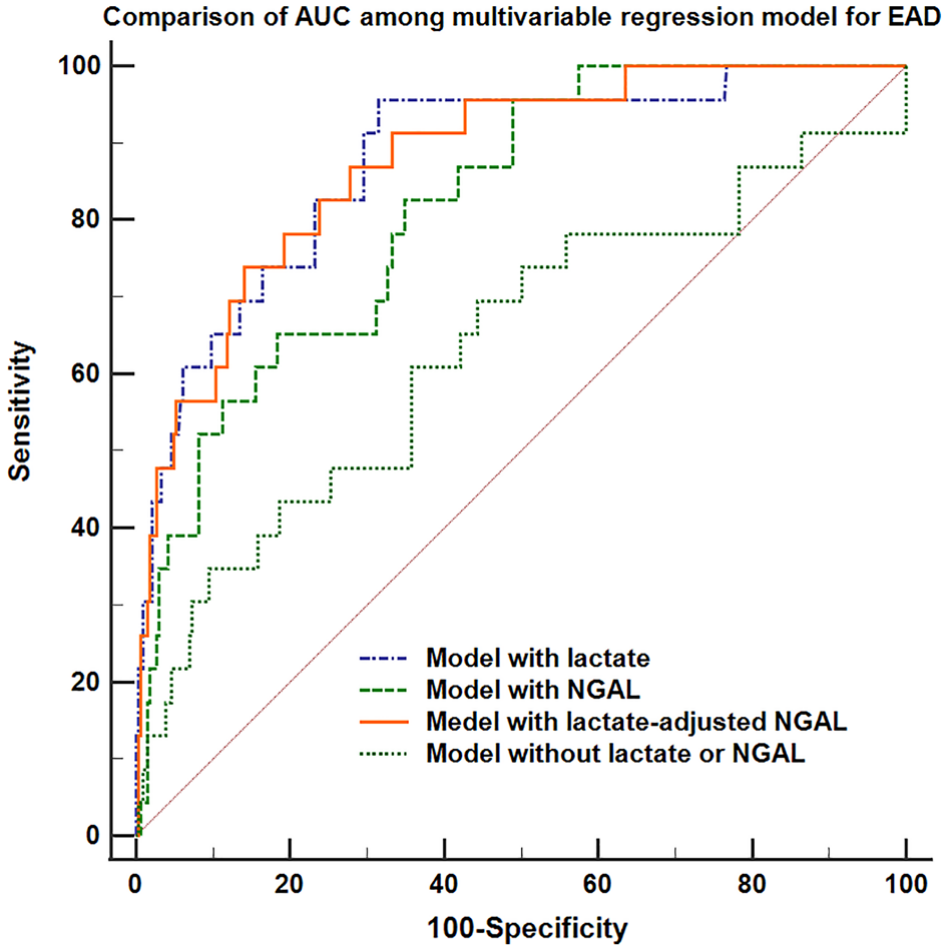
Comparison of area under the receiver operating characteristics curves between the multivariable logistic regression models including serum lactate at the end of surgery, serum neutrophil gelatinase-associated lipocalin (NGAL), and lactate-adjusted NGAL for early allograft dysfunction.

The results of multivariable logistic regression analysis for EAD are shown in Table 2. Serum NGAL level at the end of surgery was not a significant predictor for EAD (odds ratio [OR] = 1.03, 95% confidence interval [CI] 1.01–1.09, p = 0.045). Serum lactate level at the end of surgery was significantly associated with EAD (OR = 1.41, 95% CI 1.13–1.75, p = 0.002). When we replaced serum NGAL and lactate with lactate-adjusted NGAL, the variable was also a significant predictor for EAD (OR = 1.49, 95% CI 1.10–2.02, p = 0.010). When we compared the discriminative ability of the logistic regression models to predict EAD with or without serum NGAL, serum lactate, or lactate-adjusted NGAL, the model with lactate-adjusted NGAL level showed the greatest AUC compared to the model with lactate or NGAL alone or the model without (AUC of the model with lactate-adjusted NGAL: 0.88, 95% CI 0.84–0.91; vs. AUC of the model with serum lactate: 0.84, 95% CI 0.81–0.88, p = 0.048; vs. AUC of the model with NGAL: 0.82, 95% CI 0.77–0.86, p = 0.004; vs. AUC of the model without lactate or NGAL: 0.64, 95% CI 0.58–0.69, p < 0.001). Our regression model for EAD including lactate-adjusted NGAL showed a good calibration (Hosmer–Lemeshow goodness-of-fit test, χ^2^ = 10.81, p = 0.452). Figure 1 shows the comparison of the AUC of each multivariable regression model for EAD with or without lactate, NGAL or lactate-adjusted NGAL.

**Table 2:** Multivariable logistic regression analysis for early allograft dysfunction (n = 353).

Variable	Odds ratio	95% CI	P-value
NGAL at the end of surgery	1.03	1.01–1.09	0.045
or Lactate at the end of surgery	1.41	1.13–1.75	0.002
or Lactate-adjusted NGAL at the end of surgery	1.49	1.10–2.02	0.010
Baseline characteristics			
Age, years	0.98	0.94–1.03	0.373
Female	1.62	0.49–2.10	0.673
Body-mass index, kg/m^2^	1.12	0.96–1.33	0.144
Background medical status			
Hypertension, n	1.25	0.34–4.13	0.659
Diabetes mellitus, n	0.50	0.11–2.38	0.386
Alcoholic liver cirrhosis, n	1.24	0.50–1.17	0.077
Hepatocellular carcinoma, n	0.55	0.13–2.32	0.416
Cholestatic disease, n	2.35	0.56–6.79	0.242
Preoperative hemoglobin, g/dl	0.97	0.71–1.32	0.825
MELD score	1.02	0.89–1.42	0.052
Child class, versus class A			
Class B	2.29	0.33–16.16	0.405
Class C	4.85	1.13–14.72	0.040
Preoperative beta-blocker, n	1.26	0.55–2.79	0.248
Preoperative diuretics, n	2.36	0.62–4.72	0.211
Previous abdominal surgery, n	2.08	0.65–8.72	0.350
Donor/ graft factors			
Age, years	1.01	0.95–1.05	0.955
Estimated GRWR	0.87	0.26–4.82	0.104
Operation and anesthesia details			
Operation time, hour	1.01	0.94–1.06	0.178
Cold ischemic time, min	1.01	0.99–1.02	0.107
Warm ischemic time, min	1.03	0.96–1.05	0.901
Crystalloid administration, ml	1.00	1.00–1.00	0.443
Albumin administration, ml	1.00	0.99–1.03	0.838
pRBC transfusion, units	1.08	0.96–1.21	0.188
FFP transfusion, units	0.99	0.92–1.07	0.898

*MELD score* model for end-stage liver disease score, *GRWR* graft-to-recipient weight ratio, *pRBC* packed red blood cell, *FFP* fresh frozen plasma, *CI* confidence interval.

**Figure 2:**
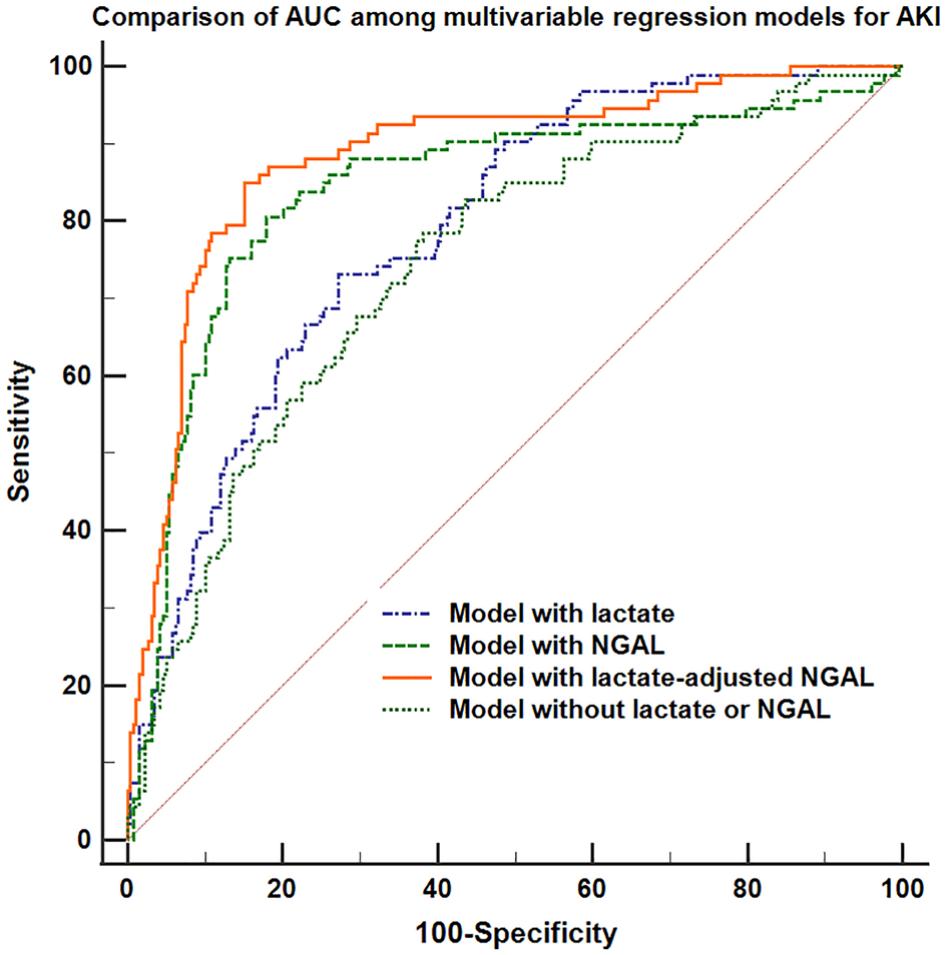
Comparison of area under the receiver operating characteristics curves between the multivariable logistic regression models including serum lactate at the end of surgery, serum neutrophil gelatinase-associated lipocalin (NGAL), and lactate-adjusted NGAL for acute kidney injury.

Table 3 shows the results of multivariable logistic regression analysis for AKI. Serum NGAL level at the end of surgery was a significant predictor for AKI (OR = 1.27, 95% CI 1.10–1.46, p = 0.001). Serum lactate level at the end of surgery was also significantly associated with AKI (OR = 1.02, 95% CI 1.02–1.03, p < 0.001). When we replaced serum NGAL and lactate with lactate-adjusted NGAL, the variable was also a significant predictor for AKI (OR = 1.33, 95% CI 1.18–1.66, p < 0.001). When we compared the discriminative ability of the logistic regression models to predict AKI with serum NGAL, serum lactate, or lactate-adjusted NGAL level, the model with lactate-adjusted NGAL level showed the greatest AUC compared to the model with NGAL or lactate alone or the model without NGAL or lactate (AUC of the model with lactate-adjusted NGAL: 0.89, 95% CI 0.85–0.92; vs. AUC of the model with lactate: 0.79, 95% CI 0.74–0.83, p < 0.001; vs. AUC of the model with NGAL: 0.84, 95% CI 0.80–0.88; p = 0.005; vs. the model without lactate or NGAL: 0.75, 95% CI 0.70–0.79, p = 0.035). Our regression model for AKI including lactate-adjusted NGAL showed a good calibration (Hosmer–Lemeshow goodness-of-fit test, χ^2^ = 13.26, p = 0.842). Figure 2 shows the comparison of the AUC of each multivariable regression model for AKI with lactate, NGAL or lactate-adjusted NGAL or the model without NGAL or lactate.

**Table 3:** Multivariable logistic regression analysis for acute kidney injury (n = 353).

Variable	Odds ratio	95% CI	P-value
NGAL at the end of surgery	1.27	1.10–1.46	0.001
or Lactate at the end of surgery	1.02	1.02–1.03	< 0.001
or Lactate-adjusted NGAL at the end of surgery	1.33	1.18–1.66	< 0.001
Baseline characteristics			
Age, years	1.03	0.98–1.05	0.484
Female	1.38	0.41–3.71	0.212
Body-mass index, kg/m^2^	1.15	1.02–1.29	0.022
Background medical status			
Hypertension, n	1.52	0.57–4.02	0.399
Diabetes mellitus, n	0.93	0.69–1.07	0.070
Alcoholic liver cirrhosis, n	1.63	0.66–2.03	0.292
Hepatocellular carcinoma, n	0.93	0.37–2.37	0.881
Cholestatic disease, n	1.93	0.51–5.22	0.331
Preoperative hemoglobin, g/dl	0.89	0.71–1.11	0.296
MELD score	1.03	1.01–1.07	0.004
Child class, versus class A			
Class B	2.89	0.87–3.63	0.084
Class C	6.06	1.10–33.3	0.039
Preoperative beta-blocker, n	4.35	1.46–12.96	0.008
Preoperative diuretics, n	0.90	0.36–2.27	0.824
Previous abdominal surgery, n	0.71	0.27–1.88	0.491
Donor/ graft factors			
Age, years	1.00	0.97–1.03	0.804
Estimated GRWR	0.69	0.19–2.61	0.589
Operation and anesthesia details			
Operation time, hour	1.06	1.01–1.22	0.050
Cold ischemic time, min	1.00	0.99–1.01	0.985
Warm ischemic time, min	1.02	0.99–1.04	0.250
Crystalloid administration, ml	1.00	1.00–1.00	0.054
Albumin administration, ml	1.00	1.00–1.00	0.578
pRBC transfusion, units	1.15	0.91–1.76	0.061
FFP transfusion, units	1.00	0.93–1.09	0.091

*MELD score* model for end-stage liver disease score, *GRWR *graft-to-recipient weight ratio, *pRBC* packed red blood cell, *FFP* fresh frozen plasma, *CI* confidence interval.

**Figure 3:**
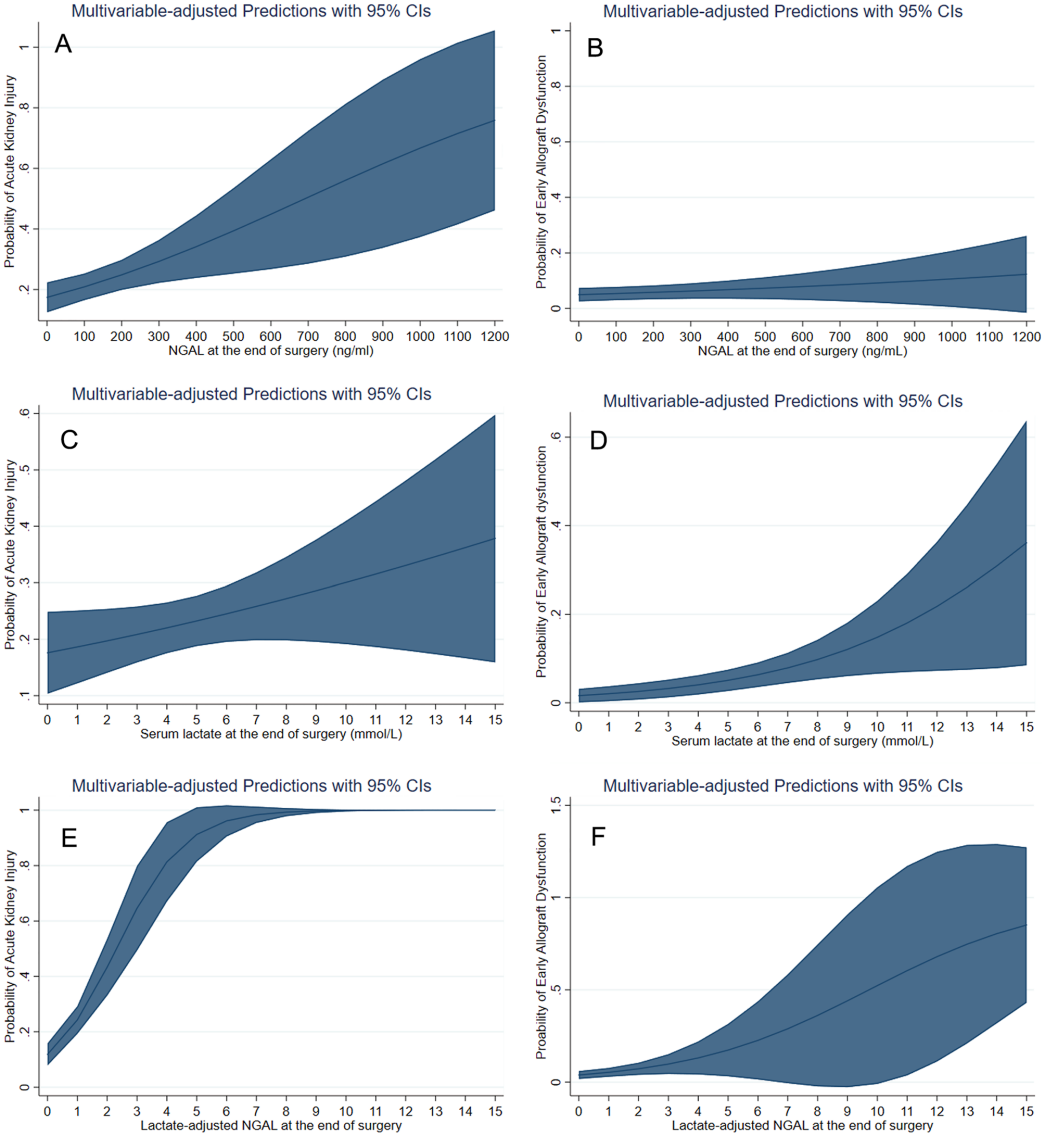
Cubic spline function curves of the multivariable-adjusted relationship between serum lactate level, serum neutrophil gelatinase-associated lipocalin (NGAL), lactate-adjusted NGAL and the risk of acute kidney injury (left column, **A**, **C**, **E**), and early allograft dysfunction (right column, **B**, **D**, **F**). Cubic spline function curve visualizes the linear or non-linear relationship between two variables and helps identify trends and patterns of the relationship. All show positive relationships but the function curve between lactate-adjusted NGAL and AKI (**E**) shows the steepest slope among all relationships.

Figure 3 is the cubic spline function curves showing multivariable-adjusted relationships of serum NGAL, lactate and lactate-adjusted serum NGAL with the risk of EAD and AKI. All show positive relationships but the function curve between lactate-adjusted NGAL and AKI shows the steepest slope among all relationships.

The optimal cutoffs of lactate-adjusted NGAL determined by Youden’s index were 125 for EAD and 191 for AKI, respectively. Using this cutoff, our study cohort was divided into two groups of high and low scores for EAD and AKI, respectively. Our propensity score matching analysis yielded 111 pairs of patients between high and low lactate-adjusted NGAL groups for EAD and 90 pairs for AKI. Supplemental Table S1 shows the comparison of baseline characteristics and perioperative parameters between the two lactate-adjusted NGAL groups before and after propensity score matching for EAD. Supplemental Table S2 shows the same comparison for AKI. Supplemental Figure S2 shows the comparison of AUC of each univariable prediction between lactate, NGAL and lactate-adjusted NGAL for EAD and AKI. The AUC of lactate-adjusted NGAL for EAD (AUC 0.83, 95% CI 0.79–0.87) is significantly greater than the AUCs of lactate (p = 0.008 vs. AUC 0.73, 95% CI 0.68–0.77) or NGAL (p = 0.013 vs. AUC 0.73, 95% CI 0.68–0.77). The AUC of lactate-adjusted NGAL for AKI (AUC 0.90, 95% CI 0.87–0.93) is significantly greater than the AUCs of lactate (p < 0.001 vs. AUC 0.55, 95% CI 0.50–0.61) or NGAL (p < 0.001 vs. AUC 0.74, 95% CI 0.70–0.79).

Supplemental Figures S3 and S4 show the histograms and covariate balance plots of our propensity score matching analysis for EAD and AKI, respectively. Table 4 shows the comparison of clinical outcomes between the two lactate-adjusted NGAL groups for EAD before and after propensity score matching. We found significant differences in the incidence of AKI, postoperative hemodialysis, EAD, one-year mortality and length of ICU stay between groups after matching. Table 5 shows the comparison of clinical outcomes between the two lactated-adjusted NGAL groups for AKI before and after matching. The incidence of AKI, postoperative hemodialysis, EAD, one-year mortality and length of hospital and ICU stay were significantly different between groups after matching.

**Table 4:** Comparison of clinical outcomes after liver transplantation between the high and low lactate-adjusted NGAL groups for early allograft dysfunction before and after propensity score matching.

	Before propensity score matching			After propensity score matching		
	Low group (n = 192)	High group (n = 161)	P-value	Low group (n = 111)	High group (n = 111)	P-value
Acute kidney injury, n			< 0.001			< 0.001
Stage 1, n	9 (4.7)	47 (29.2)		7 (16.7)	35 (31.5)	
Stage 2, n	3 (1.6)	16 (9.9)		2 (1.8)	13 (11.7)	
Stage 3, n	1 (0.5)	11 (6.8)		1 (0.9)	9 (8.1)	
Acute kidney injury stage 2 or 3, n	4 (2.1)	27 (16.8)	< 0.001	3 (2.7)	22 (19.8)	< 0.001
Postoperative hemodialysis, n	4 (2.1)	14 (8.7)	0.005	2 (1.8)	11 (9.9)	0.019
Early allograft dysfunction, n	1 (0.5)	21 (13.0)	< 0.001	1 (0.9)	13 (11.7)	0.001
In-hospital mortality, n	1 (0.5)	3 (1.9)	0.248	–	2 (1.8)	0.249
One-year mortality, n	1 (0.5)	14 (8.7)	< 0.001	1 (0.9)	11 (9.9)	0.005
Length of ICU stay, days	4 (3–4)	4 (4–7)	< 0.001	4 (3–4)	4 (3–5)	0.017
Length of hospital stay, days	24 (17–39)	45 (25–80)	< 0.001	18 (14–28)	21 (14–31)	0.099

Data are presented as the number (%) or median [interquartile range] or number (%)

*ICU* intensive care unit.

**Table 5:** Comparison of clinical outcomes after liver transplantation between the high and low lactate-adjusted NGAL groups for acute kidney injury before and after propensity score matching.

	Before propensity score matching			After propensity score matching		
	Low group (n = 233)	High group (n = 120)	P-value	Low group (n = 90)	High group (n = 90)	P-value
Acute kidney injury, n			< 0.001			< 0.001
Stage 1, n	2 (0.9)	54 (45.0)		1 (1.1)	40 (44.4)	
Stage 2, n	–	19 (15.8)			16 (17.8)	
Stage 3, n	–	12 (10.0)			11 (12.2)	
Acute kidney injury stage 2 or 3, n	–	31 (100)	< 0.001	–	27 (30.0)	< 0.001
Postoperative hemodialysis, n	3 (2.5)	15 (6.4)	0.086	3 (3.3)	11 (12.2)	0.024
Early allograft dysfunction, n	5 (2.1)	17 (14.2)	< 0.001	2 (2.2)	12 (13.3)	0.005
In-hospital mortality, n	1 (0.4)	3 (2.5)	0.116	–	2 (1.8)	0.249
One-year mortality, n	1 (0.4)	14 (11.7)	< 0.001	1 (1.1)	12 (13.3)	0.002
Length of ICU stay, days	4 (3–5)	4 (4–7)	< 0.001	4 (3–4)	4 (4–6)	0.005
Length of hospital stay, days	17 (13–26)	27 (16–46)	< 0.001	20 (14–31)	24 (16–34)	0.060

Data are presented as the number (%) or median [interquartile range] or number (%)

*ICU* intensive care unit.

## Discussion

Previous evidence for serum NGAL as a biomarker to detect AKI in patients with liver cirrhosis is mixed due to heterogeneous populations of individual studies and the different diagnostic criteria of AKI used in previous studies. The variable performance of urine NGAL to predict AKI was reported to range from 0.66 to 0.92 in terms of AUC^2,18,19^. NGAL could also predict long-term renal dysfunction after liver transplantation^20^. However, the predictive value of NGAL for AKI has mainly been reported postoperatively from the first postoperative day, which undermines the value of NGAL as an early predictor for AKI. However, when we combined NGAL with lactate at the end of the surgery, we found a significant and additional predictive value of lactate-adjusted NGAL as shown by our AUC analyses. We also revealed its prognostic value by the propensity score analysis between the high and low levels of lactate-adjusted NGAL. Therefore, lactate-adjusted NGAL level could be an early single laboratory predictor with a high discriminative ability for both EAD and AKI after liver transplantation.

We performed this study to develop a single laboratory prognostic factor to predict two important complications of EAD and AKI with high incidence and clinical implications after liver transplantation. As NGAL is one of the most popular biomarkers of AKI^1,2,18,19^ and serum lactate at the end of liver transplantation surgery has been reported to be a single laboratory predictor of EAD with high discriminative ability^3^, we attempted to combine these two laboratory values.

Previous studies determined the best cutoff of lactate at the end of surgery to be 5 mmol/L as determined by the ROC curve analysis by Youden’s index^3^. The authors demonstrated the prognostic value of serum lactate concentration for primary nonfunction, EAD and 90-day mortality by comparing the AUC of the previously-reported BAR score^21^ and BAR score with lactate. BAR-lactate score including serum lactate at the end of surgery showed a better diagnostic performance compared to the BAR score alone. Two different serum lactate groups according to the level at the end of surgery presented no difference in their preoperative baseline BAR-lactate score, highlighting the additional prognostic value provided by serum lactate at the end of surgery. Another study also reported arterial lactate concentration at the end of elective hepatectomy could warn us of the risk of severe morbidity and 90-day mortality^4^. However, the BAR-lactate score was valuable only for postoperative graft function but our lactate-adjusted NGAL level was demonstrated to have prognostic value for both graft function and AKI. AKI is a frequent complication after liver transplantation with a higher incidence compared to EAD and has also an important prognostic value^5–8^.

Previous studies reported that intraoperative and perioperative NGAL levels showed acceptable discriminative performance for AKI^1^. NGAL could predict AKI after liver transplantation early than the other biomarkers^22^. NGAL measured within 2–6 h after graft reperfusion could predict AKI after liver transplantation^23^. Biomarkers other than NGAL for AKI after liver transplantation have been reported including cystatin C, kidney injury molecule-1 and interleukin-18^1^. Cystatin C and NGAL are the most studied biomarker and could early identify AKI or the progression of AKI^1,24^. Preoperative NGAL could also predict the irreversibility of preoperative renal dysfunction in patients undergoing liver transplantation^20^.

The risk factors for EAD or AKI other than lactate or NGAL in our logistic regression analysis were mostly consistent with previous studies^5–8,11,12^. Baseline severity of liver cirrhosis^5,6,12^, preoperative low hematocrit^6,25^, and intraoperative red blood cell transfusion^6,25^ were previously reported risk factors of AKI after liver transplantation. For the risk factor of EAD, MELD score, recipient age, and cold ischemic time were reported in previous literature^7,8^, which were also significant in our analysis.

There are several important limitations in our study. Firstly, our study was a single-center retrospective study with a relatively small sample size. Sample size justification was not performed. Unknown or unmeasured bias could not be adjusted in our multivariable analysis. External validity may be limited for other populations with a different severity of baseline liver disease or different incidences of EAD or AKI. Multicenter data or prospective clinical trial is required to validate our results. Furthermore, our results should be interpreted cautiously because the average MELD score was 11 and more than 56% of our patients received transplantation due to hepatocellular carcinoma. Patients with Child classification A are most frequent in our patient cohort. Although liver transplantation is a well-established treatment for end-stage liver disease, preemptive liver transplantation is frequently performed in our country to prevent the progression of liver disease when a living donor is available. This may limit the generalizability of our study findings to other populations with different disease etiologies or disease severity. Our study results may not apply to patients with more advanced liver disease or undergoing deceased donor transplantation.

Secondly, as our retrospective cohort was relatively small, we did not divide our cohort into derivation and validation cohorts. Nonetheless, our results could be better validated by another cohort from other institutions. Thirdly, although urine NGAL level seems to have a better performance compared to serum NGAL^1,19^, serum NGAL was used in our analysis because serum NGAL test was available in our institution. As urine sample is not available for patients with end-stage renal failure, serum NGAL could not be useful for these patients with anuria. Fourthly, we could not compare the long-term survival between the two matched cohorts because our study population was collected from very recent cases.

In conclusion, our retrospective analysis revealed that the combination of serum NGAL and serum lactate at the end of surgery could be an early and reliable predictor for patient outcomes including AKI, EAD, length of ICU stay and one-year mortality. The discriminative ability to predict AKI or EAD and prognostic value of serum lactate-adjusted NGAL were revealed by our AUC and propensity score analysis. However, our results are based on the patients with low MELD scores and mostly hepatocellular carcinoma. Caution should be exercised when extrapolating our study results to other patient populations with advanced liver disease with other etiology.

## Supplementary Information

Supplementary Information.

## Data Availability

The raw data supporting the conclusion of this article will be made available by the authors, without undue reservation. The raw data will be provided if requested to the corresponding author (wonhokim@snu.ac.kr).

## Abbreviations

AUC: Area under the receiver operating characteristics curve
EAD: Early allograft dysfunction
MELD: Models for end-stage liver disease
NGAL: Neutrophil gelatinase-associated lipocalin

## References

[1] Asrani SK, Shankar N, da Graca B, Nadim MK, Cardenas A (2022). Role of novel kidney biomarkers in patients with cirrhosis and after liver transplantation. Liver Transpl..

[2] Treeprasertsuk S (2015). Urine neutrophil gelatinase-associated lipocalin: A diagnostic and prognostic marker for acute kidney injury (AKI) in hospitalized cirrhotic patients with AKI-prone conditions. BMC Gastroenterol..

[3] Golse N (2019). Arterial lactate concentration at the end of liver transplantation is an early predictor of primary graft dysfunction. Ann. Surg..

[4] Vibert E (2015). Arterial lactate concentration at the end of an elective hepatectomy is an early predictor of the postoperative course and a potential surrogate of intraoperative events. Ann. Surg..

[5] Durand F (2018). Acute kidney injury after liver transplantation. Transplantation.

[6] Park MH (2015). Clinical risk scoring models for prediction of acute kidney injury after living donor liver transplantation: A retrospective observational study. PLoS ONE.

[7] Deschenes M (2013). Early allograft dysfunction: Causes, recognition, and management. Liver Transpl..

[8] Olthoff KM (2010). Validation of a current definition of early allograft dysfunction in liver transplant recipients and analysis of risk factors. Liver Transpl..

[9] Lee HJ, Kim WH, Jung CW, Suh KS, Lee KH (2020). Different severity of clinical outcomes between the 2 subgroups of stage 1 acute kidney injury after liver transplantation. Transplantation.

[10] von Elm E (2007). Strengthening the Reporting of Observational Studies in Epidemiology (STROBE) statement: Guidelines for reporting observational studies. BMJ.

[11] Chen J (2011). Postliver transplant acute renal injury and failure by the RIFLE criteria in patients with normal pretransplant serum creatinine concentrations: A matched study. Transplantation.

[12] Utsumi M (2013). Risk factors for acute renal injury in living donor liver transplantation: Evaluation of the RIFLE criteria. Transpl. Int..

[13] Pomposelli JJ (2016). Patterns of early allograft dysfunction in adult live donor liver transplantation: The A2ALL experience. Transplantation.

[14] Thomas ME (2015). The definition of acute kidney injury and its use in practice. Kidney Int..

[15] Shin SR, Kim WH, Kim DJ, Shin IW, Sohn JT (2016). Prediction and prevention of acute kidney injury after cardiac surgery. BioMed. Res. Int..

[16] DeLong ER, DeLong DM, Clarke-Pearson DL (1988). Comparing the areas under two or more correlated receiver operating characteristic curves: A nonparametric approach. Biometrics.

[17] Ruopp MD, Perkins NJ, Whitcomb BW, Schisterman EF (2008). Youden Index and optimal cut-point estimated from observations affected by a lower limit of detection. Biom. J..

[18] Jaques DA (2019). Biomarkers for acute kidney injury in decompensated cirrhosis: A prospective study. Nephrology (Carlton).

[19] Barreto R (2014). Urinary neutrophil gelatinase-associated lipocalin predicts kidney outcome and death in patients with cirrhosis and bacterial infections. J. Hepatol..

[20] Aberg F (2014). Neutrophil gelatinase-associated lipocalin associated with irreversibility of pre-liver transplant kidney dysfunction. Clin. Transplant..

[21] Dutkowski P (2011). Are there better guidelines for allocation in liver transplantation? A novel score targeting justice and utility in the model for end-stage liver disease era. Ann. Surg..

[22] Li Y (2012). Urinary neutrophil gelatinase-associated lipocalin and L-type fatty acid binding protein as diagnostic markers of early acute kidney injury after liver transplantation. Biomarkers.

[23] Wagener G (2011). Urinary neutrophil gelatinase-associated lipocalin as a marker of acute kidney injury after orthotopic liver transplantation. Nephrol. Dial. Transplant..

[24] Belcher JM (2014). Kidney biomarkers and differential diagnosis of patients with cirrhosis and acute kidney injury. Hepatology.

[25] Kim WH (2019). Intraoperative hemodynamic parameters and acute kidney injury after living donor liver transplantation. Transplantation.

