# Supplementary material for: Serum neutrophil gelatinase-associated lipocalin and lactate level during surgery predict acute kidney injury and early allograft dysfunction after liver transplantation
Source: Sci Rep. 2023 May 27;13:8643. doi: 10.1038/s41598-023-34372-9 (PMC10224977; doi:10.1038/s41598-023-34372-9)

**Serum neutrophil gelatinase-associated lipocalin and lactate level during surgery predict acute kidney injury and early allograft dysfunction after liver transplantation**

**: Supplemental Materials**

**Supplemental Table S1**. Comparison of baseline characteristics and perioperative parameters before and after propensity score matching between the high and low lactated-adjusted NGAL groups for early allograft dysfunction.

**Supplemental Table S2**. Comparison of baseline characteristics and perioperative parameters before and after propensity score matching between the high and low lactated-adjusted NGAL groups for acute kidney injury.

**Supplemental Figure S1.** Inclusion and exclusion of our study cohort.

**Supplemental Figure S2**. Comparison of area under the receiver operating characteristics curves between the univariable prediction between serum lactate at the end of surgery, serum neutrophil gelatinase-associated lipocalin (NGAL), and lactate-adjusted NGAL for early allograft dysfunction (A) and for acute kidney injury (B).

**Supplemental Figure S3**. Histogram (upper) and covariate balance plot (lower) show the distribution of propensity score before and after matching between the two lactate-adjusted NGAL groups for early allograft dysfunction.

**Supplemental Figure S4**. Histogram (upper) and covariate balance plot (lower) show the distribution of propensity score before and after matching between the two lactate-adjusted NGAL groups for acute kidney injury.

**Supplemental Table S1**. Comparison of baseline characteristics and perioperative parameters before and after propensity score matching between the high and low lactated-adjusted NGAL groups for early allograft dysfunction.

|  | Before matching | |  | Standardized difference | After matching | |  | Standardized difference |
| --- | --- | --- | --- | --- | --- | --- | --- | --- |
| Characteristic | Low group | High group | P-value |  | Low group | High group | P-value |  |
| Sample size, n | 192 (54.4) | 161 (45.6) |  |  | 111 (50.0) | 111 (50.0) |  |  |
| Demographic data |  |  |  |  |  |  |  |  |
| Age, years | 58 (52-63) | 58 (51-64) | 0.828 | 0.11 | 58 (52-65) | 58 (53-64) | 0.746 | 0.03 |
| Female, n | 60 (31.3) | 59 (36.6) | 0.285 | 0.05 | 39 (35.1) | 40 (36.0) | 0.889 | 0.02 |
| Body-mass index, kg/m^2^ | 22.7 (20.5-26.5) | 23.4 (20.8-26.5) | 0.064 | 0.20 | 22.9 (20.8-25.1) | 23.1 (20.4-25.6) | 0.814 | 0.01 |
| Background medical status |  |  |  |  |  |  |  |  |
| Hypertension, n | 44 (22.9) | 31 (19.3) | 0.402 | 0.09 | 27 (24.3) | 27 (24.3) | 0.999 | 0.00 |
| Diabetes mellitus, n | 55 (28.6) | 46 (28.6) | 0.988 | 0.01 | 34 (30.6) | 37 (33.3) | 0.666 | 0.06 |
| Alcoholic liver cirrhosis, n | 46 (24.0) | 51 (31.7) | 0.106 | 0.17 | 27 (24.3) | 31 (27.9) | 0.541 | 0.08 |
| HBV hepatitis, n | 111 (61.7) | 69 (38.3) | 0.005 | 0.30 | 51 (45.9) | 50 (45.0) | 0.893 | 0.02 |
| HCV hepatitis, n | 13 (6.8) | 12 (48.0) | 0.803 | 0.03 | 9 (8.1) | 9 (8.1) | 0.999 | 0.00 |
| Hepatocellular carcinoma, n | 120 (62.5) | 70 (43.5) | <0.001 | 0.38 | 58 (52.3) | 57 (51.4) | 0.893 | 0.02 |
| Cholestatic disease, n | 14 (7.3) | 19 (11.8) | 0.147 | 0.15 | 11 (9.9) | 10 (9.0) | 0.819 | 0.03 |
| Preoperative hemoglobin, g/dl | 11.0 (9.4-13.0) | 9.3 (7.5-11.3) | <0.001 | 0.65 | 10.1 (8.3-12.2) | 10.1 (8.4-11.7) | 0.745 | 0.05 |
| Preoperative serum albumin level, mg/dl | 3.2 (2.8-3.8) | 2.9 (2.6-3.4) | <0.001 | 0.31 | 500 (300-700) | 500 (300-700) | 0.520 | 0.07 |
| MELD score | 9.6 (7.6-14.5) | 15.3 (9.4-24.5) | <0.001 | 0.79 | 10.9 (8.5-17.5) | 12.3 (8.8-17.0) | 0.618 | 0.08 |
| Child class, n (A/ B/ C) | 95 (49.5)/ 58 (30.2)/ 39 (20.3) | 46 (28.6)/ 51 (31.7)/ 64 (39.8) | <0.001 | 0.50 | 47 (42.3)/ 34 (30.6)/ 30 (27.0) | 41 (36.9)/ 42 (37.8)/ 28 (25.2) | 0.738 | 0.04 |
| Previous abdominal surgery, n | 55 (28.6) | 38 (23.6) | 0.274 | 0.11 | 23 (20.7) | 25 (22.5) | 0.744 | 0.04 |
| Preoperative LVEF, % | 57 (53-61) | 60 (54-63) | 0.004 | 0.30 | 58 (54-62) | 58 (54-62) | 0.661 | 0.04 |
| Preoperative beta-blocker, n | 20 (10.4) | 14 (8.7) | 0.585 | 0.06 | 13 (11.7) | 12 (10.8) | 0.832 | 0.03 |
| Preoperative diuretics, n | 47 (24.5) | 44 (27.3) | 0.542 | 0.06 | 30 (27.0) | 31 (27.9) | 0.880 | 0.02 |
| Donor/ graft factors |  |  |  |  |  |  |  |  |
| Estimated GRWR | 1.17 (0.98-1.39) | 1.23 (0.94-1.60) | 0.072 | 0.39 | 1.19 (0.99-1.41) | 1.14 (0.93-1.37) | 0.465 | 0.02 |
| Operation and anesthesia details |  |  |  |  |  |  |  |  |
| Operation time, hour | 380 (330-450) | 410 (340-488) | 0.071 | 0.16 | 390 (330-450) | 410 (343-480) | 0.342 | 0.04 |
| Cold ischemic time, min | 100 (82-132) | 120 (84-220) | 0.002 | 0.49 | 100 (84-134) | 102 (80-158) | 0.925 | 0.06 |
| Warm ischemic time, min | 32 (27-40) | 34 (28-44) | 0.097 | 0.14 | 34 (28-40) | 34 (26-44) | 0.909 | 0.03 |
| Intraoperative crystalloid administration, ml | 4300 (3400-5800) | 4800 (3650-7210) | 0.025 | 0.27 | 4650 (3600-6850) | 4700 (3600-6550) | 0.978 | 0.00 |
| Intraoperative 5% albumin administration, ml | 400 (210-680) | 500 (300-700) | 0.002 | 0.31 | 500 (300-700) | 500 (300-700) | 0.743 | 0.07 |
| Estimated blood loss, ml | 2000 (1200-4600) | 3400 (1800-6750) | <0.001 | 0.35 | 2550 (1600-5500) | 2800 (1550-4700) | 0.805 | 0.04 |

The values are expressed as the median [interquartile range] or number (%).

HBV = hepatitis B virus, HCV = hepatitis C virus, MELD score = model for end-stage liver disease score, CTP score = Child-Turcotte-Pugh score, LVEF = left ventricular ejection fraction, GRWR = graft versus recipient body weight ratio, p-RBC = packed red blood cells, FFP = fresh frozen plasma.

**Supplemental Table S2**. Comparison of baseline characteristics and perioperative parameters before and after propensity score matching between the high and low lactated-adjusted NGAL groups for acute kidney injury.

|  | Before matching | |  | Standardized difference | After matching | |  | Standardized difference |
| --- | --- | --- | --- | --- | --- | --- | --- | --- |
| Characteristic | Low group | High group | P-value |  | Low group | High group | P-value |  |
| Sample size, n | 233 (66.0) | 120 (34.0) |  |  | 90 (50.0) | 90 (50.0) |  |  |
| Demographic data |  |  |  |  |  |  |  |  |
| Age, years | 58 (51-63) | 58 (52-64) | 0.682 | 0.03 | 57 (51-57) | 58 (54-63) | 0.540 | 0.14 |
| Female, n | 72 (30.9) | 47 (39.2) | 0.120 | 0.17 | 34 (37.8) | 34 (37.8) | 0.999 | 0.02 |
| Body-mass index, kg/m^2^ | 22.8 (20.5-24.8) | 23.4 (20.9-26.7) | 0.060 | 0.22 | 23.1 (21.0-25.2) | 23.2 (20.7-26.7) | 0.861 | 0.19 |
| Background medical status |  |  |  |  |  |  |  |  |
| Hypertension, n | 51 (21.9) | 24 (20.0) | 0.681 | 0.05 | 23 (25.6) | 20 (22.2) | 0.600 | 0.15 |
| Diabetes mellitus, n | 66 (28.3) | 35 (34.7) | 0.869 | 0.02 | 30 (33.3) | 28 (31.1) | 0.750 | 0.15 |
| Alcoholic liver cirrhosis, n | 58 (24.9) | 39 (32.5) | 0.129 | 0.17 | 27 (30.0) | 23 (25.6) | 0.506 | 0.05 |
| HBV hepatitis, n | 133 (57.1) | 47 (39.2) | 0.001 | 0.36 | 34 (37.8) | 40 (44.4) | 0.363 | 0.05 |
| HCV hepatitis, n | 14 (6.0) | 11 (9.2) | 0.273 | 0.12 | 8 (8.9) | 8 (8.9) | 0.999 | 0.00 |
| Hepatocellular carcinoma, n | 141 (60.5) | 49 (25.8) | <0.001 | 0.40 | 41 (45.6) | 43 (47.8) | 0.765 | 0.15 |
| Cholestatic disease, n | 19 (8.2) | 14 (11.7) | 0.283 | 0.12 | 9 (10.0) | 10 (11.1) | 0.808 | 0.06 |
| Preoperative hemoglobin, g/dl | 10.9 (9.3-13.0) | 8.8 (7.2-10.8) | <0.001 | 0.79 | 9.8 (7.9-11.5) | 9.7 (7.6-11.3) | 0.596 | 0.10 |
| Preoperative serum albumin level, mg/dl | 3.2 (2.8-3.8) | 2.8 (2.5-3.2) | <0.001 | 0.43 | 3.0 (2.7-3.3) | 2.8 (2.5-3.3) | 0.382 | 0.13 |
| MELD score | 9.7 (7.6-14.7) | 16.9 (11.5-27.3) | <0.001 | 0.87 | 13.4 (9.0-19.5) | 15.2 (10.3-20.3) | 0.165 | 0.28 |
| Child class, n (A/ B/ C) | 115 (49.4)/ 70 (30.0)/ 48 (20.6) | 26 (21.7)/ 39 (32.5)/ 55 (45.8) | <0.001 | 0.67 | 29 (32.2)/ 35 (38.9)/ 26 (28.9) | 25 (27.8)/ 34 (37.8)/ 31 (34.4) | 0.394 | 0.16 |
| Previous abdominal surgery, n | 66 (28.3) | 27 (22.5) | 0.239 | 0.13 | 25 (27.8) | 22 (24.4) | 0.611 | 0.15 |
| Preoperative LVEF, % | 57 (53-62) | 60 (54-63) | 0.029 | 0.22 | 59 (54-63) | 60 (54-63) | 0.760 | 0.03 |
| Preoperative beta-blocker, n | 25 (10.7) | 9 (7.5) | 0.330 | 0.11 | 9 (10.0) | 8 (8.9) | 0.799 | 0.12 |
| Preoperative diuretics, n | 55 (23.6) | 36 (30.0) | 0.193 | 0.14 | 27 (30.0) | 28 (31.1) | 0.871 | 0.11 |
| Donor/ graft factors |  |  |  |  |  |  |  |  |
| Estimated GRWR | 1.18 (0.98-1.40) | 1.23 (0.94-1.73) | 0.146 | 0.33 | 1.17 (0.93-1.44) | 1.17 (0.94-1.46) | 0.712 | 0.42 |
| Operation and anesthesia details |  |  |  |  |  |  |  |  |
| Operation time, hour | 390 (335-456) | 392 (330-484) | 0.980 | 0.06 | 400 (350-490) | 390 (330-470) | 0.315 | 0.03 |
| Cold ischemic time, min | 102 (82-139) | 125 (85-240) | 0.001 | 0.49 | 106 (88-154) | 100 (77-164) | 0.487 | 0.27 |
| Warm ischemic time, min | 32 (27-40) | 35 (29-44) | 0.066 | 0.20 | 32 (28-43) | 34 (27-43) | 0.994 | 0.06 |
| Intraoperative crystalloid administration, ml | 4300 (3400-5880) | 5180 (3700-7400) | 0.003 | 0.35 | 4880 (3680-7400) | 4700 (3560-6730) | 0.749 | 0.00 |
| Intraoperative 5% albumin administration, ml | 400 (275-600) | 500 (300-800) | <0.001 | 0.43 | 500 (300-700) | 500 (300-700) | 0.983 | 0.04 |
| Estimated blood loss, ml | 2150 (1300-4550) | 4000 (2000-7700) | <0.001 | 0.43 | 3650 (2000-5850) | 3250 (1790-6310) | 0.843 | 0.06 |

The values are expressed as the median [interquartile range] or number (%).

HBV = hepatitis B virus, HCV = hepatitis C virus, MELD score = model for end-stage liver disease score, CTP score = Child-Turcotte-Pugh score, LVEF = left ventricular ejection fraction, GRWR = graft versus recipient body weight ratio, p-RBC = packed red blood cells, FFP = fresh frozen plasma.

**Supplemental Figure S1.** Inclusion and exclusion of our study cohort.


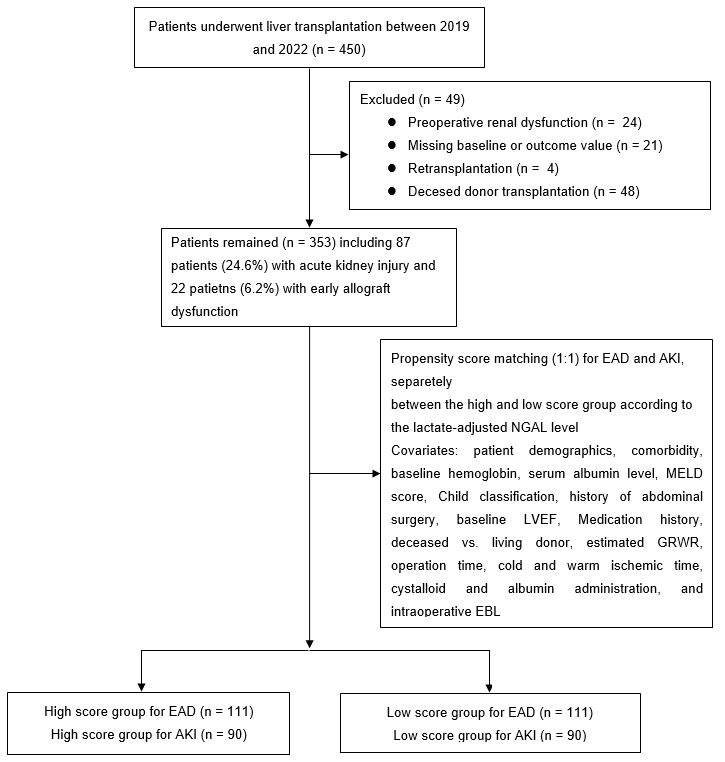


**Supplemental Figure S2**. Comparison of area under the receiver operating characteristics curves between the univariable prediction between serum lactate at the end of surgery, serum neutrophil gelatinase-associated lipocalin (NGAL), and lactate-adjusted NGAL for early allograft dysfunction (A) and for acute kidney injury (B). EAD = early allograft dysfunction, AKI = acute kidney injury.

**
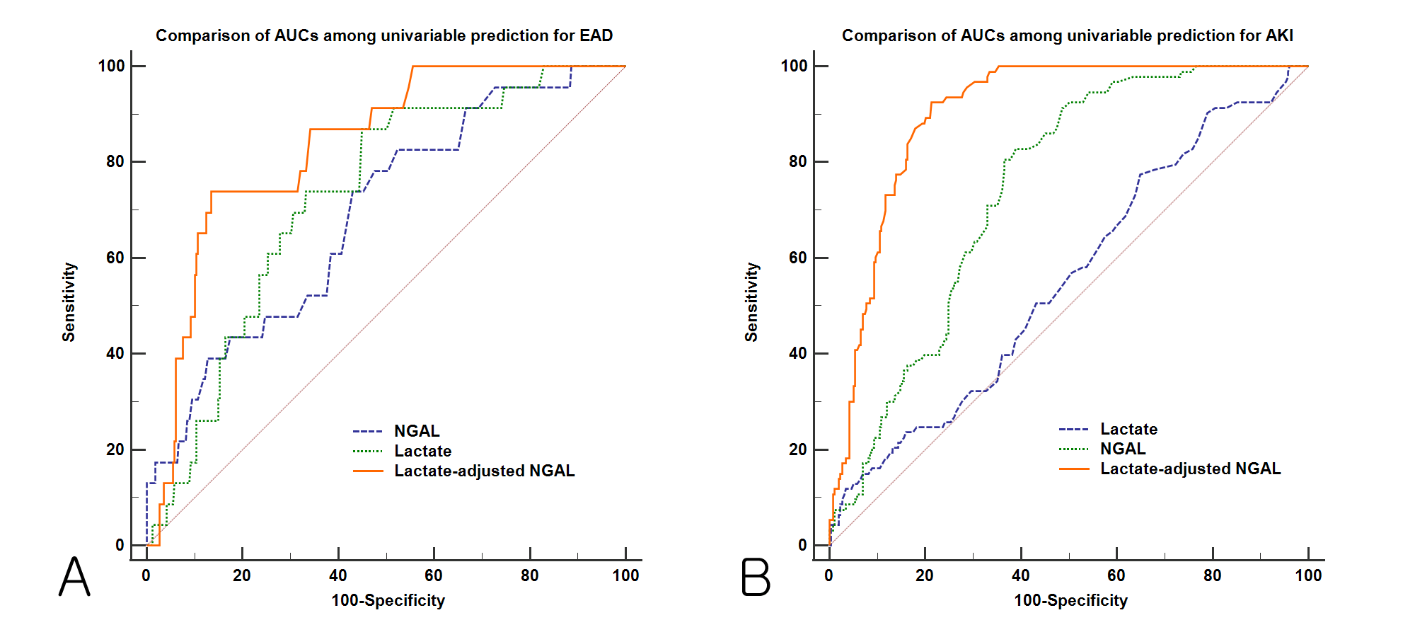
**

**Supplemental Figure S3**. Histogram (upper) and covariate balance plot (lower) show the distribution of propensity score before and after matching between the two lactate-adjusted NGAL groups for early allograft dysfunction.

Living_deceased_donor = incidence of living/deceased donor, BMI = body-mass index, HTN = hypertension, DM = diabetes mellitus, hemoglobin_preop = preoperative hemoglobin, MELD = model for end-stage-liver-disease, HCC = hepatocellular carcinoma, previous_abd_surg = history of previous abdominal surgery, op_time = operation time, RBC_tf = red blood cell transfusion during surgery, FFP_tf = fresh frozen plasma transfusion, GRWR = graft-to-recipient weight ratio.


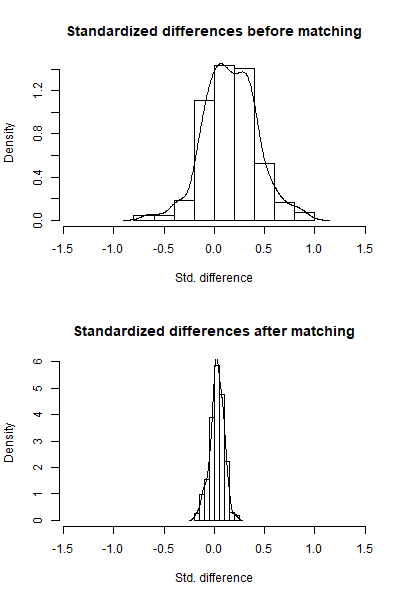

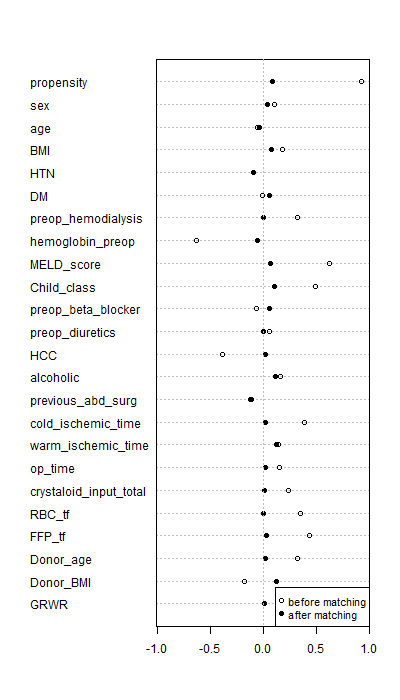


**Supplemental Figure S4**. Histogram (upper) and covariate balance plot (lower) show the distribution of propensity score before and after matching between the two lactate-adjusted NGAL groups for acute kidney injury.

Living_deceased_donor = incidence of living/deceased donor, BMI = body-mass index, HTN = hypertension, DM = diabetes mellitus, hemoglobin_preop = preoperative hemoglobin, MELD = model for end-stage-liver-disease, HCC = hepatocellular carcinoma, previous_abd_surg = history of previous abdominal surgery, op_time = operation time, RBC_tf = red blood cell transfusion during surgery, FFP_tf = fresh frozen plasma transfusion, GRWR = graft-to-recipient weight ratio.


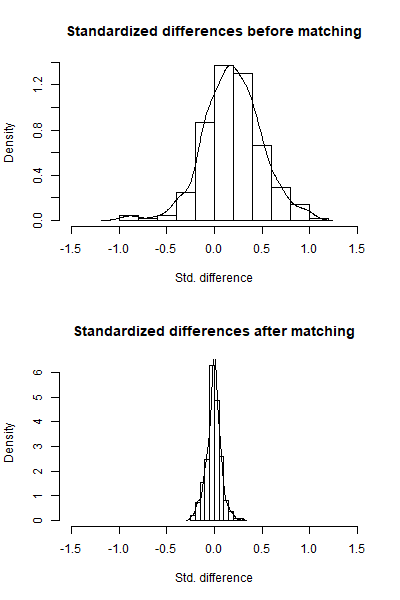

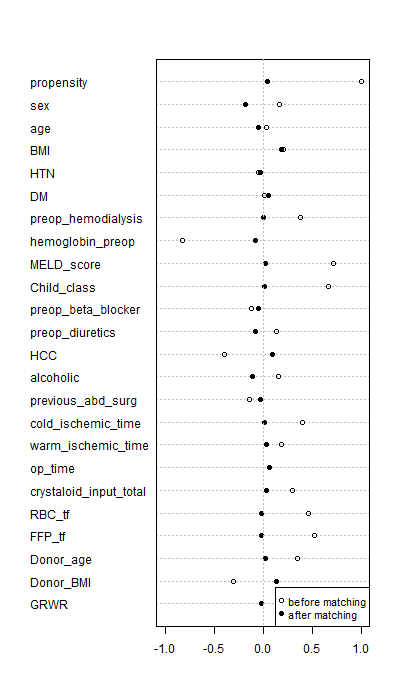

Supplement: Supplementary file 1 — Supplementary Information. [file 41598_2023_34372_MOESM1_ESM.docx]
